# Renal Artery Stenosis As Etiology of Recurrent Flash Pulmonary Edema and Role of Imaging in Timely Diagnosis and Management

**DOI:** 10.7759/cureus.7609

**Published:** 2020-04-09

**Authors:** Pradnya Brijmohan Bhattad, Vinay Jain

**Affiliations:** 1 Internal Medicine, East Tennessee State University, Johnson City, USA; 2 Radiology, James H. Quillen Veterans Affairs Medical Center, Johnson City, USA

**Keywords:** renal artery stenosis, recurrent flash pulmonary edema, duplex ultrasonography, severe hypertension

## Abstract

Renal hypoperfusion from renal artery stenosis (RAS) activates the renin-angiotensin system, which in turn causes volume overload and hypertension. Atherosclerosis and fibromuscular dysplasia are the most common causes of renal artery stenosis. Recurrent flash pulmonary edema, also known as Pickering syndrome, is commonly associated with bilateral renal artery stenosis. There should be a high index of clinical suspicion for renal artery stenosis in the setting of recurrent flash pulmonary edema and severe hypertension in patients with atherosclerotic disease. Duplex ultrasonography is commonly recommended as the best initial test for the detection of renal artery stenosis. Computed tomography (CT) angiography (CTA) or magnetic resonance (MR) angiography (MRA) are useful diagnostic imaging studies for the detection of renal artery stenosis in patients where duplex ultrasonography is difficult. If duplex ultrasound, CTA, and MRA are indeterminate or pose a risk of significant renal impairment, renal angiography is useful for a definitive diagnosis of RAS. The focus of medical management for RAS relies on controlling renovascular hypertension and aggressive lifestyle modification with control of atherosclerotic disease risk factors. The restoration of renal artery patency by revascularization in the setting of RAS due to atherosclerosis may help in the management of hypertension and minimize renal dysfunction.

## Introduction and background

Renal artery stenosis (RAS) is often associated with hypertension and ischemic nephropathy. A majority of renovascular lesions are attributed to atherosclerosis. Renovascular hypertension secondary to renal artery stenosis is a frequent curable etiology of secondary hypertension [1-2]. Renal hypoperfusion from RAS activates the renin-angiotensin system, which in turn causes volume expansion and elevation of systemic blood pressure due to the vasoactive effects of aldosterone and angiotensin II. RAS may be the etiology of end-stage renal failure in up to 20% of new dialysis patients and carries a high mortality risk in dialysis patients [1,3-4]. 

## Review

Etiology

Atherosclerosis and fibromuscular dysplasia are the most common causes of RAS. Atherosclerotic disease commonly involves the ostium and the proximal third of the main renal artery. Atherosclerosis accounts for more than 90% of RAS lesions [1-2]. Atherosclerotic renovascular lesions are common in the elderly, diabetics, patients with aortoiliac disease, hypertension, coronary artery disease, and peripheral artery disease. Fibromuscular dysplasia accounts for less than 10% of RAS. Fibromuscular dysplasia typically presents in younger females with hypertension. Fibromuscular dysplasia commonly involves the distal two-thirds of the main renal artery and its branches [1-3]. Angiography commonly demonstrates the classic ‘string of beads’ appearance and the location within the renal artery in fibromuscular dysplasia, which helps differentiate it from atherosclerotic renovascular lesions. Patients with renal fibromuscular dysplasia require magentic resonance (MR) or computed tomography (CT) angiography (CTA) of head to screen for cerebral aneurysms [4-8]. Refer to Table 1 below.

**Table 1:** Etiology of renal artery stenosis [1], [3-4], [9-12]

Summary of Causes of Renal Artery Stenosis
Atherosclerosis
Fibromuscular dysplasia
Neurofibromatosis
Vasculitis
Congenital bands
Renal artery aneurysm
Aortic or renal artery dissection
Trauma
Extrinsic compression
Ionizing radiation
Collagen vascular disease

Clinical Presentation

The characteristic findings of RAS include severe hypertension and volume overload. Recurrent flash pulmonary edema, also known as Pickering syndrome, is commonly associated with bilateral RAS [5-7]. Flash pulmonary edema is an emergent and life-threatening situation that presents with sudden respiratory distress with dyspnea, tachypnea, hypoxia, diaphoresis, and altered mentation and may eventually lead to cardiopulmonary arrest and death. Flash pulmonary edema can be precipitated by anything that leads to increased left ventricular filling pressures [5-7]. An interesting feature of recurrent flash pulmonary edema is that it may occur frequently at night due to nocturnal hypotension, and then severe renal hypoperfusion may occur from the critical narrowing of existing severe RAS lesion. This renal hypoperfusion leads to volume overload and severe hypertension seen in acute pulmonary edema due to the activation of the renin-angiotensin system [7-8,13-14].

Physical exam may reveal epigastric bruit. Diffuse atherosclerosis may be present, with evidence of atherosclerotic lesions in other vascular areas such as femoral bruits and carotid bruits. Feeble pulses may be noted [6,8-9,15-16]. There should be a high index of clinical suspicion for RAS in the setting of recurrent flash pulmonary edema, severe hypertension in patients with atherosclerotic disease, or presence of atherosclerotic risk factors such as diabetes, dyslipidemia, smoking and also in young patients with unexplained and uncontrolled hypertension [9,16-17]. Refer to Table 2 below*.*

**Table 2:** Clinical presentation of RAS RAS, renal artery stenosis; ACE, angiotensin-converting enzyme; ARB, angiotensin receptor blocker [1], [2], [4-7], [9], [12], [16], [18-23]

Classic Clinical Clues for RAS
1. Abrupt onset of hypertension: before age 30 years is commonly secondary to fibromuscular dysplasia; after age 55 years is usually from atherosclerosis
2. Resistant hypertension: previously well-controlled hypertension which becomes uncontrolled despite three-drug antihypertensive regimen including a diuretic
3. Malignant hypertension: hypertension with end-organ damage
4. Azotemia: unexplained or induced by ACE inhibitor or ARB administration
5. Unexplained asymmetric renal size: more than 1.5-cm size discrepancy between two kidneys on imaging studies
6. Unexplained atrophic kidney on imaging
7. Recurrent flash pulmonary edema despite normal left ventricular function/ejection fraction: secondary to volume overload and peripheral vasoconstriction mediated by renin–angiotensin system

Diagnostic evaluation

Laboratory examination reveals that elevated serum creatinine levels may be present in RAS. Proteinuria and bland urine sediment are usually noted on urine analysis in RAS [8,13,17-18].

Duplex ultrasonography is highly sensitive and specific for RAS, inexpensive, and easily available. It is commonly recommended as the best initial test for the detection of RAS. B-mode ultrasound imaging and Doppler frequency spectral analysis are used in arterial duplex technique [9,13,17-18]. The criteria to detect RAS include elevated peak systolic velocities, the renal-to-aortic ratio of peak systolic velocities in prerenal abdominal aorta and renal arteries, and the presence of color and spectral turbulence. Renal resistive index can be calculated by duplex ultrasound. The finding of an elevated renal resistive index is a marker of renal parenchymal disease, suggesting the lack of improvement after revascularization.

Renal resistive index = Renal parenchymal peak systolic velocity- End diastolic velocity/Peak systolic velocity [13,15,18-20].

Surveillance of renal arteries after stenting can be easily done with the help of duplex ultrasonography. Drawbacks of duplex ultrasound include difficulty in obtaining measurements from body habitus, obesity, or excess bowel gas. Another limitation is that it must be performed in experienced centers and requires operator skill. The lower sensitivity of duplex ultrasound to identify accessory renal arteries makes it difficult to identify stenosis in these vessels [18,20].

Magnetic resonance angiography (MRA) is a very useful test for the diagnosis of RAS with high sensitivity and specificity. It is a noninvasive modality and capable to generate 3D reconstructions. Limitations of MRA are that it is expensive with limited availability and may overestimate the severity of the lesions [18-19]. In the case of high-grade stenosis lesions, there is a lack of resolution, and usually, the MRI picture appears as occlusion or loss of signal. MRA is not much useful for surveillance of renal arteries after stenting as it is limited by metal artifact. MRA is less sensitive to detect fibromuscular dysplasia. As the gadolinium contrast agents have been linked to nephrogenic systemic fibrosis, the use of MRA in patients with RAS and advanced renal insufficiency becomes complicated [19,24].

CTA has high sensitivity and specificity to diagnose RAS and can generate 3D images of the renal arteries, aorta and visceral vessels. Limitations of CTA include contrast and radiation exposure [17,20]. Contrary to MRA, CTA can be used for surveillance of renal arteries after stenting to detect in-stent restenosis as it does not have a metal artifact. Thus, CTA or MRA is a useful diagnostic imaging study to detect RAS in patients where duplex ultrasonography is difficult [17,20,24-26].

Renal arteriography is the gold standard diagnostic test for RAS. It is possible to evaluate the degree of stenosis visually, to obtain hemodynamic measurements or gradients across the stenotic lesions with visualization of the main and accessory renal arteries and their branches. Limitations include the administration of nephrotoxic radiocontrast and invasive nature due to the need for intra-arterial access. The hallmark feature of the common type of fibromuscular dysplasia, “string of beads” appearance, is detectable on renal angiography [10,18,20,25,27-28]. 

If duplex ultrasound, CTA, and MRA are indeterminate or pose a risk of significant renal impairment, renal angiography is useful for a definitive diagnosis of RAS. Patients with lower-extremity peripheral artery disease or coronary artery disease have a high prevalence of RAS, and as such, obtaining a renal angiogram may be considered in patients in whom there is a high clinical suspicion for RAS and who are known to have coronary artery disease and are undergoing invasive angiography [9-10,18,20,25].

Renal scintigraphy can assess differential renal blood flow. Renal scintigraphy along with captopril renography has been used to detect RAS. Captopril renography leads to angiotensin-converting enzyme (ACE) inhibitor-induced azotemia, which is to detect differential renal perfusion expected in RAS secondary to inhibition of the maintenance of glomerular filtration rate by the renin-angiotensin mechanism in RAS. Captopril renography is not currently recommended for the screening of RAS due to lower sensitivity and specificity when compared with renal angiography [14,18,20]. Renal imaging modalities are useful to detect asymmetric renal size with a size discrepancy of more than 1.5 cm between the two kidneys, atrophic kidney suggestive of RAS [20,24].

RAS is considered hemodynamically significant when there is any stenotic lesion affecting 70% or more diameter stenosis or a lesion with 50% to 70% or more of diameter stenosis in the setting of a peak translesional pressure gradient of more than 20 mm Hg or a mean gradient of 10 mmHg or more with the pressure wire [22,25,27-28,29-31].

Treatment

Medical Management

Multidrug antihypertensive therapy for aggressive control of blood pressure is the mainstay of the treatment. Aggressive lifestyle modification with control of atherosclerotic disease risk factors is an important part of management [10,26,28-29]. The focus of medical management for RAS relies on controlling renovascular hypertension. Angiotensin receptor blockers, angiotensin-converting enzyme inhibitors, diuretics, calcium channel blockers, beta-blockers, and other antihypertensive agents are useful to manage hypertension in RAS. Angiotensin receptor blockers and angiotensin-converting enzyme inhibitors are not recommended for the treatment of hypertension in patients with bilateral RAS or those with RAS in the setting of a single kidney. In patients with bilateral RAS, most antihypertensives may lead to worsening renal function with metabolic disturbances except for beta-blockers. Angiotensin receptor blockers and angiotensin-converting enzyme inhibitors are the agents of choice to manage hypertension in the setting of unilateral RAS [26,29,31]. Target blood pressure control in patients with RAS should be according to the guidelines of the Joint National Committee on prevention, detection, evaluation, and treatment of hypertension [30-31].

Percutaneous Revascularization

The restoration of renal artery patency by revascularization in the setting of RAS due to atherosclerosis may help in the management of hypertension and minimize renal dysfunction. In patients with symptomatic RAS, revascularization may improve or stabilize renal function for almost one-year post revascularization [32-33]. According to the American College of Cardiology (ACC)/American Heart Association (AHA) peripheral artery disease guidelines, hemodynamically significant RAS in patients with recurrent unexplained pulmonary edema or congestive heart failure is the only class I indication for percutaneous renal artery revascularization. Percutaneous renal artery revascularization for hemodynamically significant RAS may be considered in the setting of unstable angina (class IIa), accelerated/malignant/resistant hypertension or hypertension with medication intolerance or in the unexplained unilateral atrophic kidney (class IIa), solitary functioning kidney or chronic kidney disease with bilateral RAS [21-22,32-34].

Renal artery stent placement is indicated for ostial atherosclerotic RAS. Congestive heart failure and chronic obstructive pulmonary disease are independent predictors of high mortality for patients undergoing renal artery stent placement. The strongest predictor of long-term mortality is baseline azotemia. Predictors of poor outcomes after revascularization for RAS are shown to be renal atrophy, renal parenchymal disease, diffuse renal arterial disease, and proteinuria more than 1 g per day [11,23,32,34].

Surgical Revascularization

Vascular surgery interventions such as surgical bypass or endarterectomy are performed for surgical revascularization in RAS [11,27,29,34]. Renal artery bypass graft for surgical revascularization of RAS in the setting of aortic aneurysmal or occlusive disease is preferred. Atherosclerotic RAS with the involvement of multiple renal arteries or early branching main renal artery is an indication for surgical revascularization. In complex situations with the involvement of segmental renal arteries, multiple renal arteries, or associated macroaneurysms surgical revascularization is preferred when anatomy is unfavorable for percutaneous revascularization [11-12,23,33].

Balloon angioplasty followed by stent placement yields higher procedural success rate compared to surgical bypass with lower restenosis. In patients with RAS due to fibromuscular dysplasia, balloon angioplasty alone without stent placement is often performed with outcomes comparable to those of stent placement [10-12,34].

## Conclusions

Recurrent flash pulmonary edema, or Pickering syndrome, is commonly associated with bilateral RAS. The constellation of clinical findings of recurrent flash pulmonary edema, severe hypertension in patients with atherosclerotic disease, or the presence of atherosclerotic risk factors should raise a suspicion of RAS. Pickering syndrome may not be present with classic clinical findings of RAS. Duplex ultrasonography is highly sensitive and specific for RAS and remains the best initial test for the detection of RAS. CTA and MRA are useful diagnostic imaging studies for the detection of RAS in patients where duplex ultrasonography is difficult.

Aggressive lifestyle modification with control of atherosclerotic disease risk factors is an integral part of the management of RAS. The focus of medical management for RAS relies on controlling renovascular hypertension. Angiotensin receptor blockers, angiotensin-converting enzyme inhibitors, diuretics, calcium channel blockers, beta-blockers, and other antihypertensive agents are useful for the management of hypertension in RAS. It is important to recognize the clinical presentation of RAS including the unusual patterns to ensure timely and directed imaging for appropriate diagnosis and management with control of symptoms and reduction of associated risks. Timely diagnosis and management of RAS are important to prevent complications and reduce morbidity and mortality. 
